# Grand multiparity in rural Cameroon: prevalence and adverse maternal and fetal delivery outcomes

**DOI:** 10.1186/s12884-019-2370-z

**Published:** 2019-07-05

**Authors:** Atem Bethel Ajong, Valirie Ndip Agbor, Larissa Pone Simo, Jean Jacques Noubiap, Tsi Njim

**Affiliations:** 1 Maternity and Surgical Department, Kekem District Hospital, Kekem, Cameroon; 2 Ibal Sub-divisional hospital, Oku, Northwest Region Cameroon; 3 Faculty of Health Sciences, University of Bamenda, Bamenda, Cameroon; 4 Department of Medicine, University of Cape Town and Groote Schuur Hospital, Cape Town, South Africa; 5 Cape Universities Body Imaging Center, University of Cape Town, Cape Town, South Africa; 6 Nuffield Department of Medicine, University of Oxford, Oxfordshire, UK; 7 Health and Human Development Research Group (2HD), Douala, Cameroon

**Keywords:** Grand multiparity, Delivery outcomes, Rural Cameroon

## Abstract

**Background:**

Grand multiparity is a major public health concern especially among developing countries and has been associated with higher risk of adverse maternal and fetal outcomes compared with women of lesser parity. There is a dearth of evidence on this subject in Cameroon, especially in the rural areas. We therefore carried out this study to document the prevalence and maternal and fetal delivery outcomes of grand multiparity in a rural Cameroonian setting.

**Methods:**

We conducted a retrospective chart review of delivery records from two health facilities (the Oku District Hospital and Kevu Integrated Health Centre) in the Oku Health District over a period of eight years. Data was entered into and analyzed using Epi-Info version 7.0.8.3. The Chi-squared or Fisher’s exact test was used to compare categorical variables. The threshold of statistical significance was set at 5%.

**Results:**

A total of 1755 delivery records met our inclusion criteria. The overall prevalence of grand multiparity was 27.0%. We found no significant difference in the rate of selected maternal and fetal delivery outcomes between grand multiparous women and those with lesser parity (*p*-value> 0.05). However, grand multiparous women were less likely to develop second-fourth degree perineal tears compared to their counterparts with lesser parity (odds ratio = 0.3, 95% confidence interval = 0.2–0.7, *p* = 0.001).

**Conclusion:**

Our study depicts a high prevalence of grand multiparous delivery in this rural community. With the exception of severe perineal tear, grand multipara and their babies are as likely to develop adverse delivery outcomes as their counterparts with lesser parity. There is also the need to enhance existing government policies on reproductive health in rural areas.

## Background

Solomon’s description of a grand multiparous woman – the “dangerous multipara” [1, 2], has been confirmed in multiple studies [2–4] in the developing setting. Over the years, varying definitions for grand multiparity have been reported in literature, ranging from women with at least four to at least eight previous deliveries [5]. However in the year 1993, the International Federation of Obstetrics and Gynecology defined a grand multiparous woman as one who has had at least five to nine prior term deliveries, and this definition has been widely adopted by authors in recent literature [5–8]. Women with 10 or more deliveries were classified as great-grand multipara [4].

Grand multiparity, especially in developing countries, has been associated with adverse maternal outcomes during pregnancy such as increased frequency of antepartum hemorrhage, placenta previa, obesity, diabetes mellitus and hypertension [2, 4, 8–10]. Even though, postpartum hemorrhage, cesarean section and malpresentation have been reported as labor and delivery complications of grand multiparity [2, 9, 10], available evidence remains inconclusive [4]. Also, grand multiparity has been associated with increased risk of preterm delivery, birth asphyxia, low birthweight and early neonatal demise [5, 8, 11]. Most of these studies have concluded on the high risk nature of grand multiparous pregnancies [3, 6, 7, 9, 10, 12].

Grand multiparity is a major public health concern especially in sub-Saharan Africa. This has been associated with a high rate of unmet contraceptive needs [13, 14], compromised or inadequate antenatal and delivery care [14], and low socio-economic status [2, 15, 16]. Developing countries are disproportionately affected by grand multiparity, with higher prevalence rates of 5.1–18.1% reported in countries like Nigeria [10]. Limited access to competent health personnel, and poor knowledge on and access to contraception aggravates the situation in rural settings [9, 10, 17]. According to the national Demographic and Health Survey (DHS) report in 2011, the average Cameroonian woman of childbearing age was a grand multipara (average fertility rate of 5.1 children per woman) [14]. This rate was even higher in rural Cameroon (6.4 as against 4.0 children per woman in urban Cameroon). About 21% of these deliveries occurred with an intergensic spacing below two years [14]. Grand multiparity is therefore a serious public health issue in Cameroon, particularly in rural zones.

An evaluation of the weight of this problem and an understanding of the maternal and fetal outcomes in a rural setting of Cameroon is indispensable as interventions aimed at reducing maternal and neonatal morbi-mortality requires this data. To the best of our knowledge, no study has been conducted to evaluate the prevalence and outcome of grand multiparity in rural Cameroon. Herein, we aimed to assess the prevalence and maternal and fetal delivery outcomes of grand multiparity in a rural Cameroonian setting.

## Methods

### Study design, duration and settings

The methods herein have been detailed in an earlier publication [18], and is part of a series of papers aimed at providing relevant data to reduce mother and child morbidity and mortality in rural Cameroon [18–20]. Briefly, we conducted a retrospective chart review of delivery records from two health facilities (Oku District Hospital and Kevu Health Center) in the Oku Health District (OHD) over a period of 8-years spanning from January 1st, 2009 to December 31st, 2016. The Oku District Hospital was managed by a single doctor, while the Kevu primary health center was managed by a nurse with no training in midwifery. Consequently, all caesarean sections were done at the district hospital. Referral in the health district is usually from the primary health care centers to the district hospital. Administratively, the OHD is located in the Bui Division of the Northwest Region of Cameroon. With an estimated population of 93,000 inhabitants, the aforementioned facilities receive a greater majority of the deliveries in this district.

### Participants and data collection

All singleton delivery records from these two facilities within the study period were targeted for this study. All delivery records lacking vital information like the number of previous deliveries were excluded. In addition, records of deliveries at a term below 28 weeks and birth weights below 1000 g (as these are considered as the threshold of fetal viability in our context), multiple pregnancies, and records with10 or more prior deliveries (considered as great-grand multipara) were excluded. To minimize bias, records of participants with caesarean section (CS) were excluded from this study. This is because CS was only conducted in one of the two health facilities where delivery records were abstracted. Furthermore, in the facility where CS was conducted, this information was only recorded as from 2014. This was highlighted in an earlier publication [18]. However, there was no association between grand multiparity and caesarean delivery (grand multiparity vs lesser parity: 3 (0.63%) vs 11 (0.86%), odds ratio (OR) = 0.7; 95% confidence interval [CI], 0.2–2.7) when these records were analyzed separately. Data was collected on sociodemographic and clinical characteristics of each mother-and-neonate pair such as: maternal age, marital status, gravidity, parity, gestational age, mode of delivery, human immunodeficiency virus (HIV) status; and the gender, 5th minute Apgar score and birth weight of their neonates. Adverse maternal outcome parameters like postpartum hemorrhage (PPH), dystocia and second-fourth degree perineal tear were recorded. Neonatal asphyxia, fetal demise and abnormal birth weights (high birthweight [HBW] and low birthweight [LBW]) were the evaluated adverse fetal outcomes.

### Statistical analysis

Data was entered into and analyzed using Epi-Info version 7.0.8.3. Variables were categorized as in Table 1 before analysis. Locally defined cut-off values of < 2600 g and > 3850 g were used to define LBW [21] and HBW [22], respectively. Means and medians were calculated for continuous variables while frequencies and their 95% CI were reported for categorical variables. The P-trend was determined using the Mann-Kendall test. With a threshold of statistical significance set at a *p*-value of 0.05, The OR and corresponding 95% CI were determined using the Chi square or Fisher’s exact test to establish associations between grand multiparity (independent variable) and selected co-variates (outcome variables).

**Table 1:** Definition of operational variables

Parity	Grand multiparity: Yes (5–9 prior deliveries) [4]; No (≤ 4 prior deliveries)
Gestational age	1. Preterm delivery: Delivery from 28 to 36 weeks of gestation 2. Term delivery: Delivery from 37 to 42 weeks of gestation 3. Post-term delivery: Delivery above 42 weeks of gestation
Apgar score at fifth minute	Neonatal asphyxia. Yes (< 7) versus No (≥ 7)
Birthweight	1. Low birthweight ≤2600 g [26] 2. Normal birthweight: 2601 – 3849 g 3. High birthweight ≥3850 [27]

## Results

**Fig. 1:**
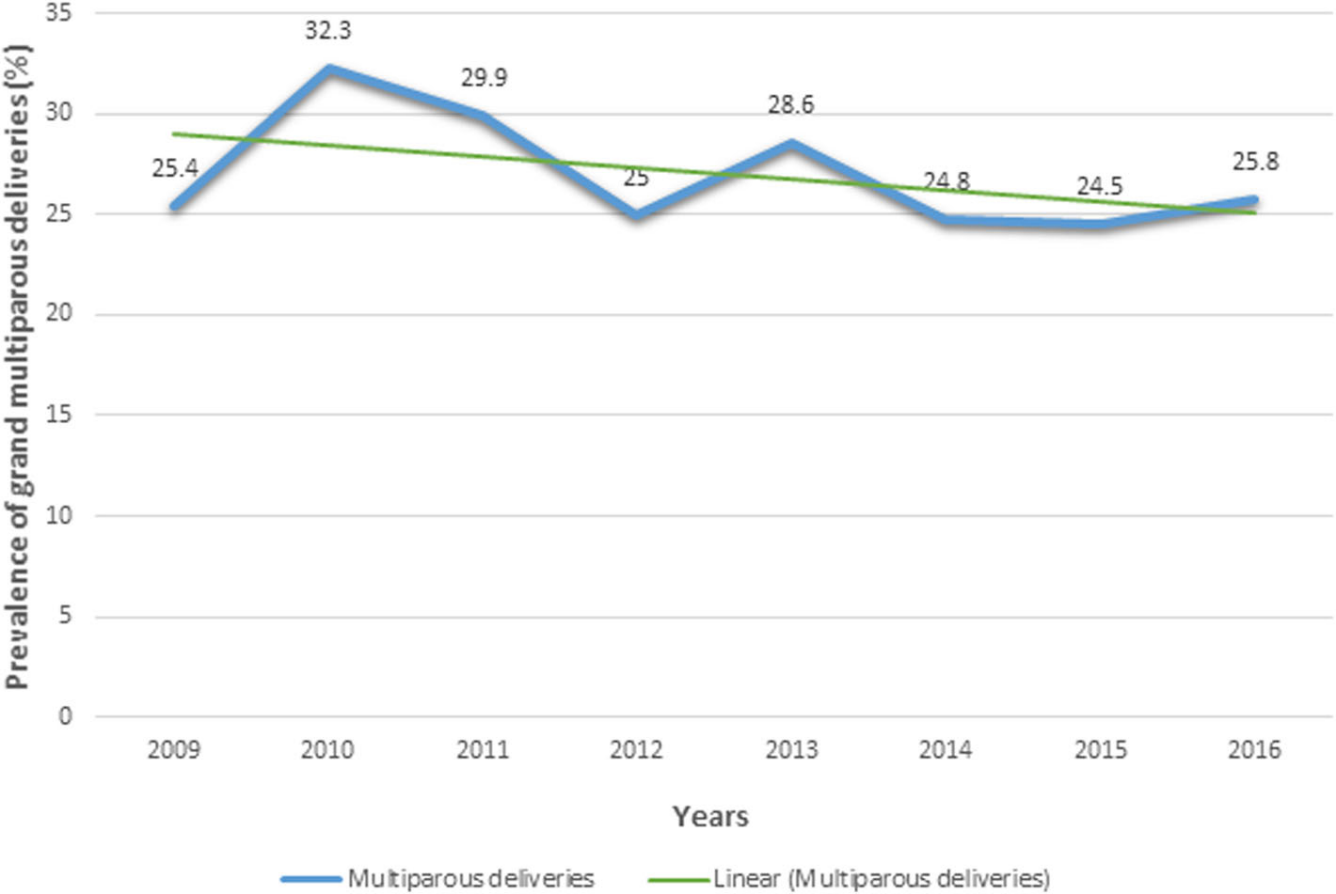
Trend in the prevalence of grand multiparous deliveries between the years 2009 and 2016 in the Oku Health District (P trend = 0.46)

Of the 1755 included deliveries, 474 occurred in grand multipara, giving an 8-year prevalence of 27.0% (95% CI = 24.9–29.1), Table 2. The mean age of the women was 33.4 ± 5.0 years and 23.2 ± 4.6 years among grand and non-grand multiparous women, respectively. The ages of our study participants ranged from 14 to 49 years. We observed a non-significantly decreasing trend in the prevalence of grand multiparous deliveries (P trend = 0.46) over the study period, Fig. 1. Over 90% of grand multipara in our study were married. Among the 1751 cases with a reported maternal HIV status, 4.5% were positive, Table 2.

**Table 2:** Sociodemographic and clinical characteristics of the study population, Oku Health District, 2009–2017

Variable	Grand multiparity		Total N (%) = 1755 (100.0%)
	Yes, *n* = 474 (27.0%)	No, *n* = 1281 (73.0%)	
Maternal age (Years)			
Mean (SD)	33.4 (5.0)	23.2 (4.6)	
Median (IQR)	33.5 (30.5–37.5)	23.5 (19.0–26.5)	
Age group (Years)			
[< 20]	0 (0.0)	361 (28.2)	361 (20.6)
[20 to 29]	94 (19.8)	798 (62.3)	892 (50.8)
[30–39]	328 (69.2)	121 (9.4)	450 (25.6)
[≥40]	52 (11.0)	1 (0.1)	53 (3.0)
Marital status			
Single	39 (8.3)	393 (30.8)	432 (24.7)
Married	434 (91.7)	882 (69.2)	1315 (75.3)
Maternal HIV status (*n* = 1752)			
Positive	19 (4.0)	59 (4.6)	78 (4.5)
Negative	454 (96.0)	1220 (95.4)	1673 (95.5)
Gestational age (*n* = 1608)			
Term	259 (59.9)	744 (63.3)	1003 (62.4)
Preterm	153 (35.3)	372 (31.7)	525 (32.6)
Post term	21 (4.8)	59 (5.0)	80 (5.0)
Birthweight (*n* = 1725)			
LBW	47 (10.1)	122 (9.7)	169 (9.8)
Normal birthweight	383 (82.6)	1071 (84.9)	1453 (84.3)
HBW	34 (7.3)	68 (5.4)	102 (5.9)
Gender of neonate			
Male	243 (51.5)	641 (50.2)	884 (50.6)
Female	229 (48.5)	635 (49.8)	864 (49.4)

HIV: Human immunodeficiency virus; N: frequency; SD: standard deviation; IQR: interquartile range; LBW: low birthweight; HBW: high birthweight

There was no significant difference in adverse maternal outcomes such as postpartum hemorrhage, dystocia, cesarean section, and premature rupture of membranes, among grand multiparous women compared to their counterparts with a lower parity (*p*-value > 0.05). However, grand multiparous women were less likely to develop second-fourth degree perineal tears compared to women with lesser parity (OR = 0.3 [95% CI = 0.2–0.7], p-value = 0.001; Table 3). When evaluated for fetal outcomes (HBW and LBW, neonatal asphyxia, stillbirth and pre- and post-term deliveries), there were no statistically significant differences between grand multiparous women and those with lesser parity, Table 4.

**Table 3:** Comparison of maternal outcomes between grand multiparous deliveries and delivery of lesser parity, Oku Health District, 2009–2016

Outcome	Grand multiparity		OR (95% CI)	*p* – value
	Yes, *n* = 474	No, *n* = 1281		
Postpartum haemorrhage, *n* (%)				
Yes	2 (0.4)	5 (0.4)	1.1 (0.2–5.6)	0.603
No	472 (99.6)	1276 (99.6)		
PROM, *n* (%)				
Yes	5 (1.1)	15 (1.2)	0.9 (0.3–2.5)	0.537
No	469 (98.9)	1266 (98.8)		
Caesarean delivery, *n* (%)				
Yes	3 (0.6)	11 (0.9)	0.7 (0.2–2.7)	0.770
No	471 (99.4)	1270 (99.1)		
Dystocia, *n* (%)				
Yes	2 (0.4)	5 (0.4)	1.1 (0.2–5.6)	0.603
No	472 (99.6)	1276 (99.6)		
Second - fourth degree perineal tear, *n* (%)				
Yes	9 (1.9)	68 (5.3)	0.3 (0.2–0.7)	0.001*
No	465 (98.1)	1213 (94.7)		

OR: Odd’s ratio; CI: Confidence interval; n: frequency; PROM: Premature rupture of membranes; *significant *p* value

**Table 4:** Comparison of fetal outcomes between grand multiparous deliveries and deliveries of lesser parity, Oku Health District, 2009–2016

Outcome	Grand multiparity	OR (95% CI)		*p* – value
	Yes, *n* = 474	No, *n* = 1281	(73.0%)	
Birthweight (BW)				
Low BW, *n* (%)	47 (10.1)	122 (9.7)	1.1 (0.8–1.5)	0.712
Normal BW, *n* (%)	383 (82.6)	1071 (84.9)	1	
High BW, *n* (%)	34 (7.3)	68 (5.4)	1.4 (0.9–2.2)	
Neonatal asphyxia (5th min Apgar)				
Yes, *n* (%)	9 (2.0)	45 (3.6)	0.5 (0.3–1.1)	0.117
No, *n* (%)	465 (98.0)	1236 (96.4)		
Stillbirth				
Yes, *n* (%)	8 (1.7)	15 (1.2)	1.4 (0.6–3.4)	0.477
No, *n* (%)	464 (98.3)	1264 (98.8)		
Term of delivery				
Preterm, *n* (%)	153 (35.3)	372 (31.7)	1.2 (0.9–1.5)	0.163
Term, *n* (%)	259 (59.9)	744 (63.3)	1	
Post-term, *n* (%)	21 (4.8)	59 (5.0)	1.0 (0.6–1.7)	

OR: Odd’s ratio; CI: Confidence interval; n: frequency

## Discussion

We sought to determine the prevalence and adverse maternal and fetal delivery outcomes in our study population. Firstly, we observed a very high prevalence of grand multiparity in our study (27%) with a non-significantly decreasing trend between the years 2009 and 2016. Secondly, grand multipara were less likely to develop second – fourth perineal tears than women with a lower parity. No other significant difference in adverse maternofetal delivery outcomes was found among grand multiparous women and those of lower parity.

This high prevalence of grand multiparity is similar to the 26.5% prevalence rate reported by Idoko et al in a group of pregnant women in Gambia [23]. Also, the investigators of the DHS 2011 highlighted a high prevalence of grand multiparity in rural Cameroon [14]. Indeed, in the Northwest Region of Cameroon where the present study was conducted, the fertility rate in the year 2011 was higher in rural than urban areas (6.4 versus 4.4 children per woman of childbearing age) [14]. This is principally related to the desire for larger family sizes for more labor in farming, mistakes and failed contraception, death of another child, and desire for children of a particular gender [23]. The myth among some rural Cameroonian communities that having more wives and children reflects how wealthy you are, pushes most men in these communities to demand more children from their wives who have little or no control on their sexual and reproductive health. Most of these families find it difficult to train these children through school, or at least supply their basic needs. These children tend to drop out of school and do odd jobs to cater for themselves. Younger girls, majority of whom have a poor knowledge on contraception, will engage in sexual relations with older boys who can provide for them, thereby increasing the rate of sexually transmitted infections, adolescent pregnancies, socioeconomic hardship and poverty. In fact, an earlier study conducted in this rural community revealed a high prevalence of adolescent pregnancy, reflecting on the burden of grand multiparity in this community [18]. Such high prevalence of grand multiparity in rural Cameroon could partly explain why Cameroon failed to attain the objectives of the Millennium Development Goal (MDG) five that was set for the year 2015 [24], and remains a potential threat to the attainment of the sustainable development goal (SDG) three [25] if appropriate measures are not taken to strengthen the application of existing government-based programs on sexual and reproductive health in rural Cameroonian communities. Furthermore, a high prevalence of grand multiparity indirectly prevents the attainment of SDGs 1, 2 and 4 [25].

However, studies conducted in other developing countries like Pakistan, Turkey and Qatar have reported lower than 5% rates of grand multiparity [12, 17, 26]. The fact that these studies were all carried out in urban zones could explain these low prevalence rates. The cultural, socioeconomic and educational backgrounds of these samples are different from that in rural Cameroon. According to the DHS report in 2011, only 24.1% of rural Cameroonians had acquired at least a secondary education and up to 44.3% could neither read nor write.

The rate of second-fourth degree perineal tears was significantly lower in grand multiparous women compared to women of lesser parity. This is probably due to the already extended and flaccid vaginal and perineal muscles due to repeated deliveries. Our result was concordant with those of Eskandar [27] and Riskin-Mashiah [28] where lesser parity was found to be significantly associated with higher risk of severe perineal tears.

Grand multipara were as likely to develop complications such as postpartum hemorrhage, premature rupture of membranes, dystocia, and cesarean section compared to women of lesser parity. A systematic review of grand multiparity in 2006 [4] and a Nigerian survey [29] in 2002 showed that there was no significant difference in postpartum hemorrhage between grand multiparous women and those of lesser parity. Plausible explanations are the multiple training sessions health personnel are submitted to and sensitizations on the application of standard protocols for management of the third stage of labor.

We found no significant difference in adverse fetal outcomes between grand multiparous women and women of lesser parity, Table 4. Even though, there is strong evidence linking abnormal birthweights (HBW and LBW) to increasing parity [1, 3, 8, 10], we found no significant association between abnormal fetal weights and grand multiparity. Our findings corroborated with those of Omole et al [29] who reported no significant difference in abnormal birthweights (LBW and HBW) between grand multiparous women and their counterparts with a lesser parity. Heterogeneity in the definitions of abnormal birthweight used in our study could explain this discrepancy as we used locally generated cut-off values to define LBW and HBW. Also, we found no significant association between neonatal asphyxia, stillbirth and grand multiparity. This finding was similar to those of Alamin et al from Sudan [2]. Other authors have reported no significant association between neonatal asphyxia and increasing parity [2, 3]. Contrastingly, Yves et al highlighted grand multiparity as a major determinant of neonatal asphyxia and perinatal death [4].

Our study was designed with some limitations, which we believe should be taken into consideration during the global appraisal of the findings presented herein. First, the retrospective study design gave us limited control over the quality of data entered into the registers. Nevertheless, we hope the quality of the data used for this study was ameliorated by strict study selection criteria. The low rates of adverse maternal outcomes among the different subgroups is a possible limitation to the statistical power of our analysis. In addition, this study was not designed to evaluate adverse antepartum and postpartum outcomes such as antepartum hemorrhage, placenta previa, eclampsia, obesity, and diabetes mellitus which have been shown to complicate grand multiparous pregnancy [2, 4, 9, 10]. Hence, the findings herein are insufficient to disqualify pregnancy in a grand multipara as a high-risk pregnancy. However, these findings resonant with existing literatures disqualifying grand multiparity as a determinant of adverse maternal and fetal outcome during labor and delivery. This study is the first to reveal the prevalence of grand multiparity in rural Cameroon, and with a large sample size, this study provides a contemporaneous picture on the prevalence and outcome of grand multiparity in this rural community.

## Conclusion

About one in four women in this sub-division is a grand multipara. There has been a non-significantly decreasing trend in the prevalence of grand multiparity over an eight-year period. Also, compared to women of lesser parity, grand multipara were less likely to develop perineal tears, and grand multiparity did not increase the odds of adverse maternal and fetal outcomes during delivery. More studies, preferably with a prospective design and large enough sample sizes, are recommended throughout the national territory to validate these preliminary findings and evaluate adverse antenatal and postnatal complications of grand multiparous deliveries in Cameroon. Enhancement of existing government policies on reproductive and sexual health are needed to curb potential socioeconomic burden of grand multiparity in rural areas.

## Data Availability

The datasets generated and/or analyzed during the current study are available from the corresponding author on reasonable request.

## Abbreviations

CI: Confidence interval
DHS: Demographic and Health Survey
HBW: High birthweight
LBW: Low birthweight
MDG: Millennium development goal
OHD: Oku District Hospital
OR: Odd’s ratio
SDG: Sustainable development goals
